# Species-specific drought impacts on black and white rhinoceroses

**DOI:** 10.1371/journal.pone.0209678

**Published:** 2019-01-16

**Authors:** Sam M. Ferreira, Nikki le Roex, Cathy Greaver

**Affiliations:** 1 Scientific Services, South African National Parks, Skukuza, South Africa; 2 Institute for Communities and Wildlife in Africa (iCWild), Department of Biological Sciences, University of Cape Town, Cape Town, South Africa; University of Tasmania, AUSTRALIA

## Abstract

Unrelenting poaching to feed the illegal trafficking of rhinoceros (rhino) horn remains the principle threat to the persistence of south-central black and southern white rhino that live in the Kruger National Park (Kruger), South Africa. Other global environmental change drivers, such as unpredictable climatic conditions, impose additional uncertainties on the management and persistence of these species. The drought experienced in Kruger over the 2015/2016 rainy season may have affected rhino population growth and thus added an additional population pressure to the poaching pressure already occurring. Under drought conditions, reduced grass biomass predicts increased natural deaths and a subsequent decrease in birth rate for the grazing white rhino. Such variance in natural death and birth rates for the browsing black rhino are not expected under these conditions. We evaluated these predictions using rhino population survey data from 2013 to 2017. Comparisons of natural deaths and birth rates between pre- (2013/2014 and 2014/15), during- (2015/2016) and post-drought (2016/2017) periods in Kruger showed increased natural mortality and decreased births for white rhino, but no significant changes for black rhino, supporting our predictions. As a result, despite reduced poaching rates, the total mortality rate of white rhino remains significantly higher than the birth rate. Decreased poaching, decreased natural deaths and no apparent drought effects in black rhino resulted in a lower total mortality rate than the estimated birth rate in 2017. Active biological management and traditional anti-poaching initiatives together therefore represent the most likely way to buffer the impacts of decreased population growth through climate change and wildlife crime on the persistence of rhinos.

## Introduction

Overharvesting of biological resources is a key driver of global environmental change [1]. Overharvesting can occur within ecosystems, biomes, landscapes, individual species or populations. On a large scale, poachers extensively overharvest particular species to supply the ever-increasing illegal wildlife trade [2]. As a result, wildlife trafficking is one of the key threats to the persistence of many species [3], and reduces the legal benefits derived from wildlife commodities [4]. Poaching of African rhinoceroses (rhino) to supply the illegal wildlife trade with rhino horn escalated within South Africa from 2008 [5], with at least 2936 rhinos killed from 2011–2015 in the Kruger National Park (Kruger) alone [6].

Climate change is another driver of global environmental transformation [1] and have consequences for species [7]. Changing environmental conditions can also influence rhino population dynamics. For instance, white rhino calf survival is associated with rainfall experienced in the previous two years [8]. Droughts are defined as climatic events or periods with significantly less rainfall compared to the average rainfall in that area [9], and can significantly impact animal survival [10]. Drought events typically have more rapid, intense impacts on grasses and herbaceous vegetation, and less impact on trees and browse condition in the short-term [11–13]. Increased animal mortalities during a drought are primarily associated with limited food availability rather than a lack of drinking water [14–16].

Increased mortality is, however, not the only potential population-level consequence of a drought. Disruptions of population features such as age and sex structures, as well as birth and recruitment rates, may also occur. For instance, African elephant (*Loxodonta africana*) cows require good foraging to achieve and maintain body condition for conception and pregnancy [17]. Consequently, across several African elephant populations, deviations in population age structure are associated with rainfall in the preceding two years before birth [18].

Kruger is a stronghold for both the south-central black rhino (*Diceros bicornis minor*; hereafter black rhino) and southern white rhino (*Ceratotherium simum simum*, hereafter white rhino). Poaching, however, has disrupted the dynamics of both species within the park [8], resulting in no significant increase in population size for black or white rhino since 2011 [6]. In addition to the poaching onslaught, Kruger experienced a severe drought over the 2015/2016 rainy season [19]. This has raised concerns that the Kruger rhino populations suffered two separate, intense population pressures in recent years–drought impacts as well as poaching mortalities. Species-specific dietary requirements, however, suggest that the relative impact of drought pressure may differ between species. Given that the short-term impact of drought is higher on grasses [11–13], the grazing white rhino [20] is likely to be more susceptible to mortality under drought conditions than the browsing black rhino [21,22]. In addition, because white rhino cows are likely to experience reduced body condition as a result of limited food availability during drought periods [22,23], they may also have reduced conception rates during this time. If droughts do not result in browse availability changes in the short-term, then black rhino cows should have relatively little change in body condition and conception during a drought period and thus birth rates the following year.

The 2015/2016 drought experienced in Kruger provided a unique opportunity to evaluate predictions regarding the relative impact of drought on white and black rhino. We estimated the population size of both rhino species in Kruger during September 2017. We also extract proxies for birth and death rates for both rhino species from field surveys from 2013 to 2017. We compare the birth and death rates before, during and after the 2015/16 drought. We predict that the white rhino decline noted before the drought [6] will be accentuated, with increased mortality and reduced fecundity, regardless of poaching rates. For black rhino, we expect the impact of drought on fecundity and mortality to be negligible and therefore predict that only poaching is likely to impact the population dynamics of this species. These differences would impose unique challenges to the management of white and black rhino, particularly for authorities seeking to achieve species-specific conservation targets [24–25] in the face of future climate change.

## Material and methods

### Study area

The Kruger National Park (19485 km^2^, 24°0′41″S 31°29′7″E) is comprised of African savannas in the Lowveld region of South Africa. Our study focused on the southern region of the park where the majority of rhinos are found [6]. The southern area experienced the drought during the 2015/2016 rainy period (293 mm, 73% of 11 stations in southern Kruger had rainfall below station-specific 0.1 percentiles). Annual rainfall for 2013/2014 (693 mm) and 2014/2015 (425 mm) were within the range of the long-term mean (575 mm, 0.025 percentile = 318 mm, 0.975 percentile = 1017 mm, n = 24, rainfall data from 1982 to 2006; SANParks, unpublished data, http://dataknp.sanparks.org/sanparks/), much higher than that recorded for 2015/2016. During the 2016/2017 rainy season, Kruger managers recorded rainfall within the range of the long-term average again (625 mm, none of the stations had rainfall below station-specific 0.1 percentiles, while one station had rainfall above the station-specific 0.9 percentile).

### Field surveys

The surveys during September 2017 followed the same approach as previous surveys [6, 8]. Observers counted both black and white rhinos on 489 randomly placed blocks of 3 km x 3 km in size within the southern region of Kruger. Counts conducted from a Jet Bell Ranger helicopter flown at 65 knots and an altitude of 45m allowed observers to note rhinos within a 200 m strip on each side of the flight path within a block. In addition to the pilot, there were three observers, one of whom also served as a scribe. Cybertracker software (Cybertracker Software (Pty) Ltd) using a Juno Trimble Unit (Trimble Navigation Limited) allowed the scribe to efficiently record the number of individual rhino noted for each sighting. Observers also noted the age and sex of each observed rhino using age-assignment criteria based on relative body sizes [26–27].

### Data analyses

We determined the number of rhinos that died in the year preceding each survey by collating mortality data in a 12 month period (survey interval year) starting on 16 September and ending on 15 September the following year. Mortality data (SANParks, unpublished data, http://dataknp.sanparks.org/sanparks/) included the date at which a carcass was detected as well as the age of the carcass estimated at the time of detection based on the level of decomposition and scavenging. The cause of death, including natural (defined as when horns were still present and forensic investigations found no evidence of poaching), poached or unknown, was also recorded. This allowed us to estimate the date of the actual death of each individual, from which we could then define the number of rhino that died within each defined survey interval year. We included mortality data from 16 September 2012.

To define natural death rates, we included carcasses for which the cause of death was recorded as natural. While both natural and poached deaths will be underestimates given the effect of imperfect detection on fatality estimates [28], we assume carcass detection rate to be the same irrespective of species or cause of death. Thus we use the recorded minimum counts for both natural and poaching deaths as proxies. We extracted population estimates for both black and white rhino during September each year for 2013 to 2016 [6]. Death rates were the number of natural deaths in a survey year as a fraction of the population estimate at the start of that year ($\frac{d_{\underset{t-1,t}{\to }}}{{\hat{N}}_{t-1}}$, where $d_{\underset{t-1,t}{\to }}$ is the number of natural deaths from 16 September in year *t*-1 to 15 September in year *t*, and ${\hat{N}}_{t-1}$ the population estimate during September of year *t*-1). The confidence intervals for population estimates allowed us to also define a confidence interval for death rates during each year.

To define birth rates, we extracted the age and sex assignments for each of the surveys during 2014 to 2017 (SANParks unpublished data, http://dataknp.sanparks.org/sanparks) [6]. We determined population recruitment by calculating the fraction of rhino individuals in the A and B age-class [26–27] in our sample each year. A (<6 months old) and B (6 months to 1 year old) age-classes comprise individuals that were born and survived during the year since the previous survey. Using the population estimate in year *t*, we could define the number of rhino in the A and B age-class categories at the time of the survey in year *t*. The number of rhino estimated during year *t*-1 (${\hat{N}}_{t-1}$) produced the new rhino that we noted in year *t*. A proxy for birth rates were thus the combined number of rhino in the A and B age-class categories noted in year *t* (*n*_(*A*+*B*),*t*_) expressed as a fraction ($\frac{n_{(A+B),t}}{{\hat{N}}_{t-1}}$) of the estimated population during September in year *t*-1. The confidence intervals for population estimates also allowed us to define a confidence interval for birth rates during each year.

To test our predictions of drought effects on mortality, we defined survey interval years 2013–2014 and 2014–2015 as pre-drought conditions, and 2015–2016 as during drought conditions. Because our pre-drought data include two survey years, we used Monte Carlo simulation approaches [29] and drew 20000 values from the statistical distributions of estimated death rates defined by their confidence intervals extracted for the 2013–2014 and 2014–2015 survey interval years. For iterations, we averaged the values for 2013–2014 and 2014–2015. We then defined a statistical distribution of pre-drought death rates by counting the frequency of estimates within 100 bins of equal sizes between the largest and smallest death rate within the 20000 randomly drawn estimates. A similar process for 2015–2016 allowed us to obtain a statistical distribution of death rates during drought conditions. In this case we did not need to do averaging across two survey interval years.

We used the same approach as used for death rates, to define the statistical distribution of birth rates for pre- and post-drought (2016–2017) conditions. Both rhino species have long gestation periods (black rhino: ≈15 months; white rhino: ≈16 months) [30], and consequently any changes in birth rate should be reflected 1–2 years following a drought. We concluded that changes were significant if the 95% confidence intervals (defined by the range between the 0.025 and 0.975 percentiles) [31] of the birth and death rate distributions did not overlap between the relevant periods defining drought conditions.

For the 2017 population estimate, we used the same analytical method as for the previously published 2013 to 2016 surveys [6,8]. These used Jolly’s estimator [32] to obtain landscape-specific and overall estimates. Analyses accounted for bias using estimated availability bias defined by relationships [6] between rhino visibility [8], vegetation cover [33] and Enhanced Vegetation Index (EVI) [34] on a block at the time. Observer bias came from estimates made during a previous black rhinoceros survey [35]. Detectability bias was negligible as the size of observation strips were narrower than those used by previous studies [36]. We concluded significant changes if any of the 95% confidence intervals of the years in questions did not overlap.

## Results

The number of rhinos that were born and died due to natural causes varied across the five years of our study. Poachers killed the fewest number of black (Table 1) and white (Table 2) rhinos during the last survey interval year (2016–2017) in our study. In addition to deaths, authorities removed a large number of white rhinos during the 2015–2016 survey interval year (Table 2).

**Table 1:** Summary of population estimates [6,8] as well as the number of mortalities, management removals and births noted for black rhino in Kruger National Park from September 2012. Values in brackets denote 95% confidence intervals.

BLACK RHINO			Mortality			Management Removal		Fecundity
Year	Estimate	Survey year	Poached	Natural	Unknown	Lethal	Non-lethal	Born
2013	415 (343–487)	2012–2013	39	10	0	0	0	47 (39–55)
2014	310 (249–371)	2013–2014	29	12	1	0	7	22 (18–26)
2015	383 (313–453)	2014–2015	39	15	0	0	2	36 (29–42)
2016	407 (349–465)	2015–2016	41	11	3	1	0	28 (24–32)
2017	507 (427–586)	2016–2017	17	5	0	2	0	33 (28–38)

**Table 2:** Summary of population estimates [6,8] as well as the number of mortalities, management removals and births noted for white rhino in Kruger National Park from September 2012. Values in brackets denote 95% confidence intervals.

WHITE RHINO			Mortality			Management Removal		Fecundity
Year	Estimate	Survey year	Poached	Natural	Unknown	Lethal	Non-lethal	Born
2013	8968 (8394–9564)	2012–2013	683	79	25	0	50	905 (847–966)
2014	8619 (8001–9290)	2013–2014	705	107	28	1	99	920 (822–954)
2015	8875 (8365–9337)	2014–2015	853	94	4	2	90	772 (728–812)
2016	7235 (6649–7830)	2015–2016	632	127	40	1	202	692 (636–749)
2017	5142 (4759–5532)	2016–2017	513	118	21	13	13	345 (319–371)

**Fig 1:**
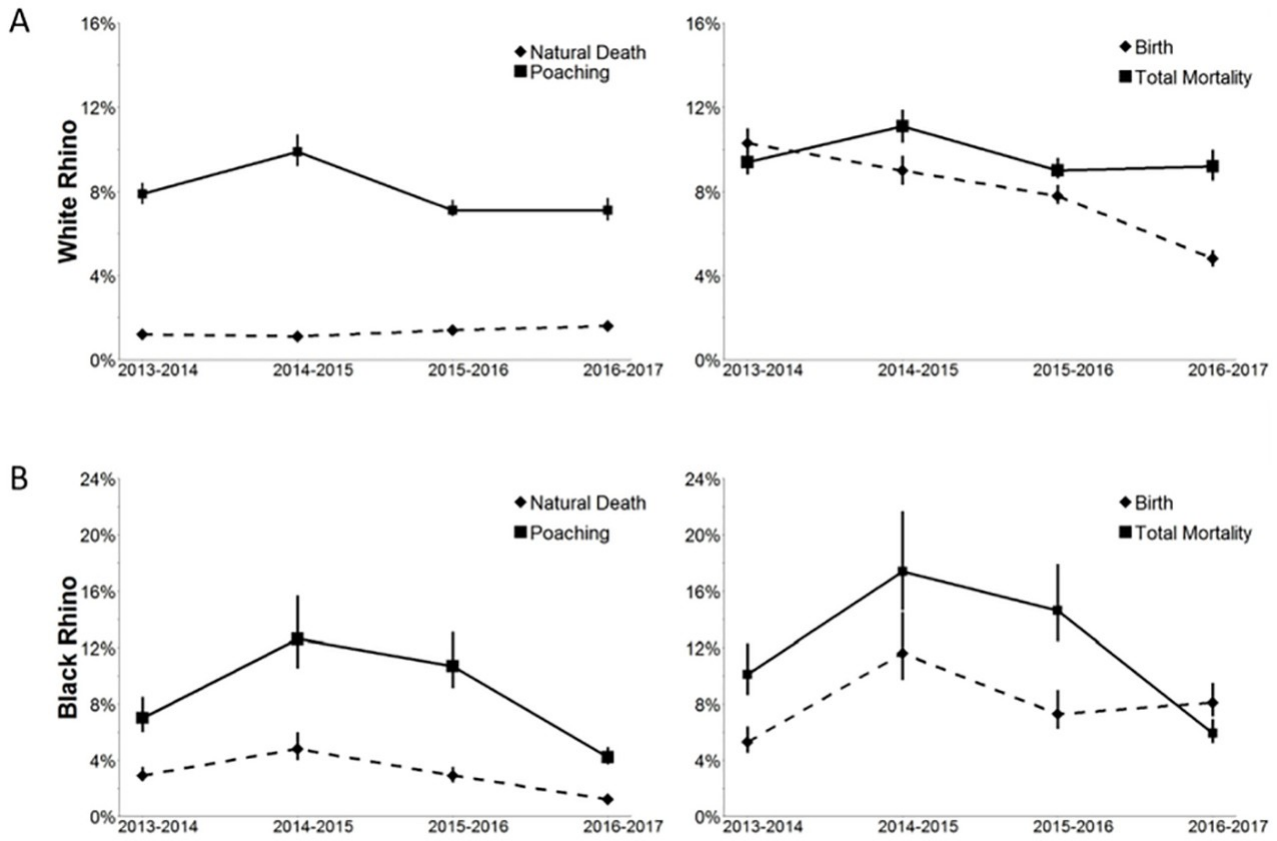
**(A)** White rhino natural death and poaching rates (left) and total mortality and birth rates (right); **(B)** Black rhino natural death and poaching rates (left) and total mortality and birth rates (right).

**Fig 2:**
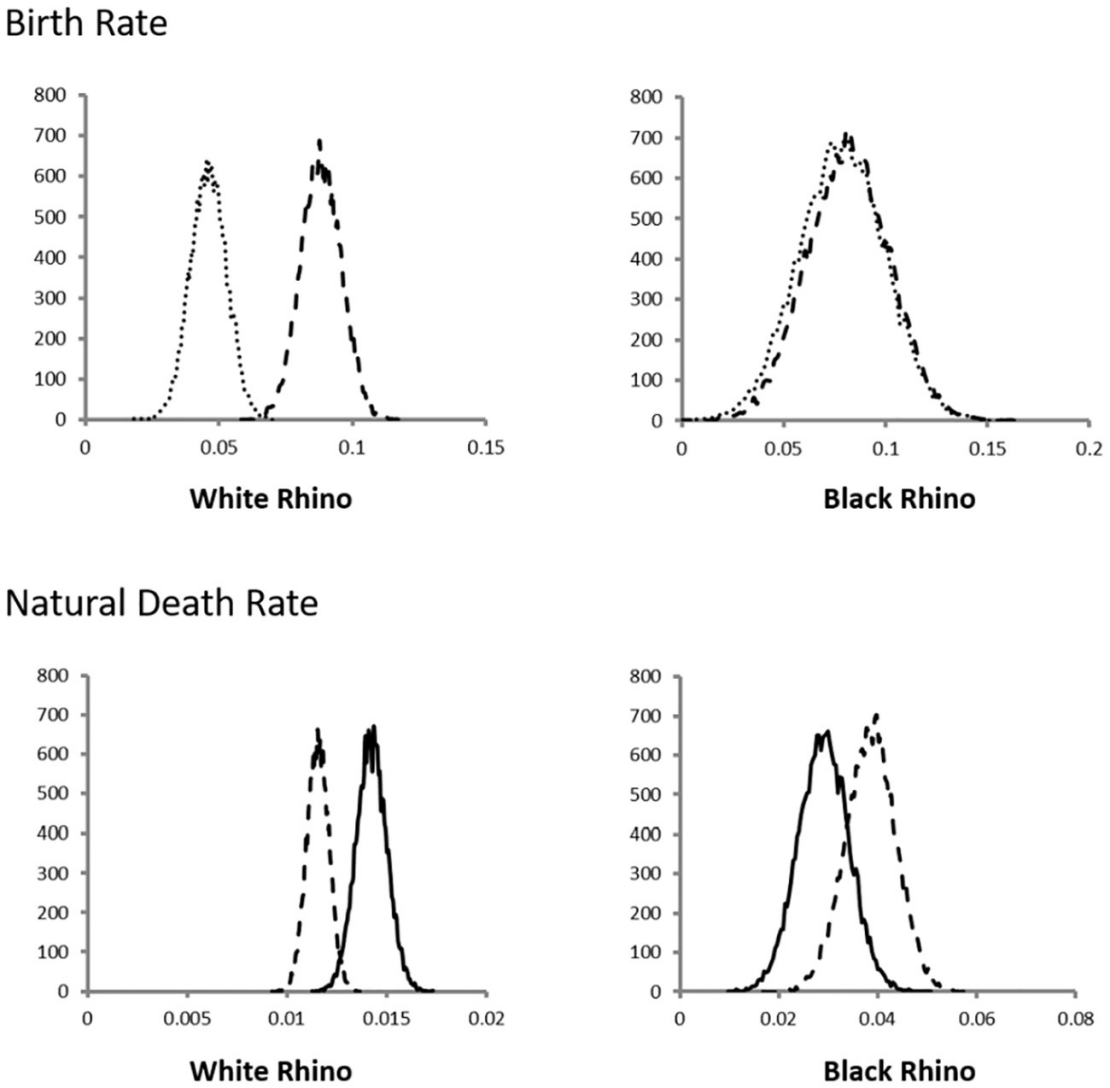
The distributions of the estimated proxies for birth rate and natural death rate for white and black rhino before (broken line), during (solid line) and after (stippled line) drought conditions in southern Kruger National Park. See text for details on estimation and definition of statistical distributions.

Poaching rates for white rhino varied from 6.7% (CI: 6.1–7.2%) to 9.3% (CI: 8.7–10.0%), with the highest rate noted during the 2014–2015 survey interval year (Fig 1). Natural death rate varied from 1.1% (CI: 1.0–1.2%) to 1.6% (CI: 1.5–1.7%). The 95% confidence intervals for natural white rhino deaths in pre-drought conditions did not overlap with those noted during the drought, indicating that natural death rates were significantly higher during the drought (Fig 2). Similarly, 95% confidence intervals of birth rates noted during pre-drought conditions did not overlap with those noted after the drought; indicating that birth rates were significantly lower after drought conditions (Fig 2). Thus despite the effectiveness of anti-poaching, the change in vital rates in response to the drought resulted in the white rhino population estimate in September 2017 (5142, 95% CI: 4759–5532; Fig 3) being significantly lower than the 7235 (CI: 6649–7830) estimated for the previous year.

**Fig 3:**
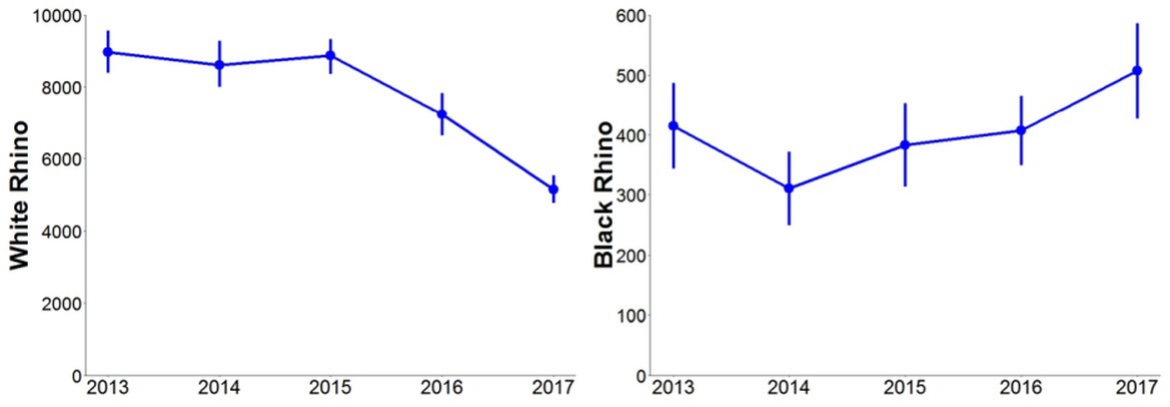
Population trends in white and black rhino in the Kruger National Park from 2013–2017.

The results for black rhino did not show the same patterns as white rhino. Poaching rates for black rhino varied from 4.1% (CI: 3.5–4.6%) to 11.5% (CI: 9.5–13.5%), with the highest poaching rate observed in the 2014–2015 survey interval year (Fig 1). Natural causes contributed 29% and 28% to deaths during 2013–2014 and 2014–2015 respectively, and only 21% and 23% during 2015–2016 and 2016–2017 respectively, when poaching rates were lower. For black rhinos, confidence intervals for death rates and birth rates overlapped between the comparative pre-, during- and post-drought periods (Fig 2). Consequently, the black rhino population estimate in September 2017 was 507 (CI: 427–586), significantly higher than the 310 estimated in 2014 (CI: 249–371). This suggests that the black rhino population has increased over the last 3 years (Fig 3).

## Discussion

The 2015/2016 drought in Kruger increased the natural death rate and decreased the birth rate of white rhino. These drought effects disrupted white rhino population dynamics and we noted a significant decline in population size despite constant poaching rates. Black rhino did not exhibit drought-related population consequences, as expected given their browsing diet and reproductive life-history [30]. Poaching thus remains the largest threat to the persistence of black rhino in Kruger.

The grazing diet and reproductive life-history of white rhino [30] suggested that the species may experience significant drought-related effects on birth and death rates. The detectable decline of white rhino numbers during 2016 [6] continued with a further significant decrease in 2017, despite the consistent poaching rate maintained over that period. Increased natural deaths during the drought and reduced births in the post-drought periods suggest that the drought impacts on white rhino negated the gains made by stabilising the poaching rate during this time.

In contrast to white rhino, the black rhino population estimate for 2017 overlapped with the previous 2015 and 2016 estimates. However, the 2017 estimate was significantly higher than the 2014 estimate [6], suggesting a gradual population increase over those three years. Black rhino experienced a reduction in poaching during 2015–2016, which decreased even further during 2016–2017. A large fraction of black rhinos live in central areas within Kruger further from boundaries [35], and with increased anti-poaching pressure, poachers may be penetrating the central areas less frequently. Consequently, they would be more likely to encounter white rhinos when entering Kruger across boundaries. Drought conditions did not result in increased natural mortality or decreased birth rates in black rhino.

Density-dependent population responses may contribute to the decreasing natural death trend seen for black rhino. Black rhino natural death rates did not increase in the drought years. In fact, natural death rates may have decreased following the drought period although we did not detect a statistically significant effect. Decreased death rates may result from additional factors such as reduced intra-specific density, rather than a direct consequence of the drought. For instance, in some cases population growth rate is reduced as local black rhino density increases [37]. Such density-dependence in population growth is often associated with density-related mortalities for black rhinos [38]. The potential decline in natural death rate that we observed in Kruger followed the peak in poaching of black rhinos. Localised reductions in black rhino density as a result of high poaching may have resulted in decreased fighting mortalities.

Several constraints, however, may influence the magnitude of our predictions and subsequent observations. For instance, water and food availability influence how white rhino use a landscape. Rainfall across Kruger is not uniform [39] and thus neither is grass availability. Kruger management begun closing boreholes in the mid-1990’s while neighbouring properties retained them [40], thus also creating spatial variation in water availability. Movements of white rhino in response to drought-induced variance in food and water resources could thus influence estimates of population size and hence also death and birth rates. Given the large white rhino population size in Kruger, however, we anticipate that exceptionally large numbers of white rhino would have to move across large scales in or out of Kruger to significantly affect our estimates of death and birth rates. This is unlikely, and as no movements of this scale were recorded during the study period, spatial variance in resource availability most likely had little effect on our estimates.

An additional limitation may exist with the assignment of black and white rhino calves to A and B age-classes [26–27]. Error-induced high frequencies of A and B age-classes would inflate the birth rate proxies. However, making errors when assigning individual rhino as A and B classes is likely to be lower than for the C to F age-classes because of the reduced development of horns in the A and B classes [26–27], and thus age assignment errors most likely have a negligible impact on our birth rate estimates. Density-dependent effects may also realize through Allee effects [41], when population growth rates positively associate with density. When densities decrease, populations decline faster either because predation pressure increases due to reduced vigilance [42], or reduced mating opportunities resulting in fewer births [43]. The large population size of white rhinos recorded, however, negates reduced mating opportunities and corresponding birth rates. For black rhinos, we recorded similar birth rates before and after the drought which also means similar birth rates at different population densities. Allee effects through reduced mating opportunities are thus negligible for both species.

The combined impact of poaching and drought effects on white rhino resulted in a substantial difference between the 2016 and 2017 estimates. A contributing factor to this difference may lie with carcass detection. Carcass detection is not perfect [28], and our observations of dead rhinos are thus estimates of minimum poaching and natural death rates only. There may also be additive effects associated with the death of dependant calves; detecting small, dependent calves that die when their mothers are poached is difficult due to the small body size and faster decomposition rate. In addition, when poachers kill a cow, all her future calves are also lost. For large mammals, adult female survival and fecundity exhibit low and moderate annual variation, respectively [44]. Rhino populations are thus likely to be sensitive to the cascading consequences of increased variability in adult female survival as a function of poaching. These hidden demographic risks may realize in substantial declines over time. As poaching has been rampant since 2012, the substantial decline in white rhinos may be partly a result of these compound effects.

The conservation implications for both rhino species are significant. The reduced poaching rate of black rhino in 2017 and the absence of drought-related impacts have led to stable or gradually increasing numbers in Kruger. With birth rate currently exceeding total mortality, continued protection from poaching may be sufficient to support the population growth of black rhino. Conversely, the drought during 2015/2016 imposed additional challenges on white rhino conservation [6]. During the 2016–2017 survey interval year, birth rate did not exceed total mortality or the poaching rate. The drought impact on mortality and birth rates, combined with continued poaching, has reduced the white rhino population size to an extent that the resilience of the population may be compromised. Recovery of the white rhino population may now take much longer due to the additional consequences of the drought. Innovative biological management initiatives that focus on increasing population growth would be beneficial to both species–reducing natural mortalities where possible in black rhino and increasing birth rate in white rhino. This will be particularly important for white rhino under changing environmental conditions.

Increased rhino protection initiatives have reduced the number of rhino poached across South Africa and in Kruger since 2016. Poacher activity, however, continued to increase in Kruger during the 2016–2017 survey year compared to previous years (SANParks, unpublished data, http://dataknp.sanparks.org/sanparks/). Elsewhere, multi-pronged approaches resulted in desirable rhino conservation outcomes [45]. Similarly, a combination of active biological management [24–25] and traditional anti-poaching initiatives represents the most likely way to buffer the impacts of decreased population growth through climate change and wildlife crime on the persistence of rhinos in Kruger.
